# Bronchiectasis, Low IgG Levels and Lack of Vaccination are Risk Factors for Covid-19 Hospitalization in X-linked Agammaglobulinemia – A Retrospective Multicenter Study

**DOI:** 10.1007/s10875-025-01962-3

**Published:** 2025-11-15

**Authors:** Caroline Stenlander, Hannes Lindahl, Emelie Wahren-Borgström, Christoph B. Geier, Anna Sediva, Børre Fevang, Cinzia Milito, Cláudia Varandas, Cristina Roca-Oporto, Federica Pulvirenti, Isabel Hodl, Ivana Malkusova, Jacques G. Rivière, Jiri Litzman, Jolan E. Walter, Leif G. Hanitsch, Olaf Neth, Pavlina Kralickova, Rahim Miller, Serena Shaffren, Susana L. Silva, Terese Katzenstein, Timi Martelius, Urs C. Steiner, C. I. Edvard Smith, Klaus Warnatz, Peter Bergman

**Affiliations:** 1 Department of Laboratory Medicine, Clinical Immunology, Karolinska Institutet, Stockholm, Sweden; 2 Department of Clinical Immunology and Transfusion Medicine, Karolinska University Hospital, Stockholm, Sweden; 3 Department of Clinical Neuroscience, Karolinska Institutet, Stockholm, Sweden; 4 Department of Infectious Diseases, Karolinska University Hospital, Stockholm, Sweden; 5 Division of Immunology, Faculty of Medicine and Health Sciences, University Medicine Oldenburg, Oldenburg, Germany; 6 Institute of Medical Genetics, Faculty of Medicine and Health Sciences, University Medicine Oldenburg, Oldenburg, Germany; 7 Center for Chronic Immunodeficiency (CCI), Medical Center, University of Freiburg, Freiburg, Germany; 8 Department of Immunology, 2nd Faculty of Medicine, Charles University and Motol University Hospital, Prague, Czech Republic; 9 Section of Clinical Immunology and Infectious Diseases, Division of Surgery and Specialized Medicine, Oslo University Hospital, Oslo, Norway; 10 Institute of Clinical Medicine, Faculty of Medicine, University of Oslo, Oslo, Norway; 11 Department of Molecular Medicine, Sapienza University, Rome, Italy; 12 ULS Santa Maria, Hospital de Santa Maria, Lisbon, Portugal; 13 Clinical Unit of Infectious Diseases, Microbiology and Parasitology, Institute of Biomedicine of Seville (IBiS), University Hospital Virgen del Rocio, Seville, Spain; 14 Reference Centre for Primary Immune Deficiencies, Sapienza University Hospital Policlinico Umberto I, Rome, Italy; 15 Division of Rheumatology and Immunology, Department of Internal Medicine, Medical University of Graz, Graz, Austria; 16 Department of Immunology and Allergology, Faculty of Medicine, Faculty Hospital in Pilsen, Charles University in Prague, Pilsen, Czechia; 17 Infection and Immunity in Pediatric Patients Research Group, Vall d’Hebron Institut de Recerca (VHIR), Barcelona, Catalonia Spain; 18 Pediatric Infectious Diseases and Immunodeficiencies Unit, Hospital Infantil I de La Dona Vall d’Hebron, Vall d’Hebron,Barcelona Hospital Campus, Barcelona, Catalonia Spain; 19 Jeffrey Modell Diagnostic and Research Center for Primary, Immunodeficiencies, Barcelona, Catalonia Spain; 20 Department of Clinical Immunology and Allergology, St. Anne’s University in Brno, Brno, Czechia; 21 Faculty of Medicine, Masaryk University, Brno, Czechia; 22 Division of Pediatric Allergy & Immunology, Department of Pediatrics, University of South Florida, Tampa, FL USA; 23 Division of Pediatric Allergy & Immunology, Department of Medicine, Johns Hopkins All Children’s Hospital, St. Petersburg, FL USA; 24 Institute for Medical Immunology, Charité Universitaetsmedizin, Berlin, Germany; 25 BIH Center for Regenerative Therapies (BCRT), Humboldt University and Free University, Berlin, Germany; 26 Paediatric Infectious Diseases, Rheumatology and Immunology Unit, Institute of Biomedicine of Seville (IBiS), University Hospital Virgen del Rocio, University of Seville/CSIC, Seville, Spain; 27 Institute of Clinical Immunology and Allergy, University Hospital Hradec Kralove, Hradec Kralove, Czech Republic; 28 Faculty of Medicine in Hradec Kralove, Charles University, Hradec Kralove, Czech Republic; 29 Faculdade de Medicina, Universidade de Lisboa, Lisbon, Portugal; 30 GIMM – Institute for Molecular Medicine, Lisbon, Portugal; 31 Department of Infectious Diseases, Copenhagen University Hospital, Rigshospitalet, Copenhagen, Denmark; 32 Inflammation Center, Infectious Diseases, HUS Helsinki University Hospital and University of Helsinki, Helsinki, Finland; 33 ERN-RITA Core Center, RITAFIN, Helsinki, Finland; 34 Department of Clinical Immunology, University Hospital Zurich, Zurich, Switzerland; 35 Karolinska ATMP Center, Karolinska Institutet, Karolinska University Hospital, Stockholm, Sweden; 36 Department of Rheumatology and Clinical Immunology, Medical Center, University of Freiburg, Freiburg, Germany

**Keywords:** X-linked agammaglobulinemia, SARS CoV-2, Covid-19, Bronchiectasis, Vaccination, IgG trough levels

## Abstract

X-linked agammaglobulinemia (XLA) is caused by loss-of-function variants in Bruton’s tyrosine kinase, leading to absence of circulating B lymphocytes and inability to produce antibodies. Despite the fear that patients with XLA would be at high risk for severe infection when the novel virus SARS-CoV-2 emerged in the society with low pre-existing immunity, most patients with XLA did not suffer from severe disease. However, some patients were critically affected. Factors associated with hospitalization in patients with XLA remain poorly described. Thus, we designed a study to determine risk factors associated with hospitalization due to Covid-19 in patients with XLA. Data was collected from 17 sites in Europe and the US, comprising *n* = 81 patients, with hospitalization due to SARS-CoV-2 infection in 14 patients. Nearly 17% of patients with XLA required hospitalization due to Covid-19, but only 3 patients had ventilatory support. After correcting for the effect of the date of infection during the early pandemic, univariate and multiple logistic regression analysis showed that preexisting bronchiectasis and lower IgG serum trough levels (< 8 g/L) before infection were associated with an increased risk for hospitalization, with a high rate of superinfection. The lack of vaccination seemed to contribute to this risk, and ambulatory patients had higher amounts of CD4^+^ T cells before infection compared to hospitalized patients. Thus, our data suggests a need for IgG trough levels above 8 g/L, especially in patients with bronchiectasis, to protect patients with XLA during viral infections such as Covid-19 and reduce morbidity due to superinfections.

**Supplementary Information:**

The online version contains supplementary material available at 10.1007/s10875-025-01962-3.

## Introduction

X-linked agammaglobulinemia (XLA) is a monogenic disease caused by a defect in Bruton’s tyrosine kinase gene (*BTK*) [1]. Due to the severe impairment of the pre B cell receptor (BCR) and BCR signaling in BTK deficient B cells, very few B cells mature beyond the pre B cell stage and enter circulation with the resulting lack of endogenous antibody production [2, 3]. Currently, more than 1000 unique pathogenic variants in the *BTK* gene are described, with some hypomorphic variants allowing for residual BTK protein expression and detectable but low immunoglobulin (Ig) levels [4]. Patients typically present with bacterial and viral infections of the respiratory and gastrointestinal tract already during early childhood [5]. Further, bronchiectasis, which is a common complication in XLA, increases the risk for frequent bacterial and viral respiratory tract infections [6, 7].

In the end of year 2019 a new corona virus, Severe Acute Respiratory Syndrome coronavirus 2 (SARS-CoV-2), was identified in humans in Wuhan, China, causing disease ranging from mild respiratory airway infection to severe acute respiratory syndrome with extrapulmonary organ failure [8–10]. It has been shown that male gender, higher age and chronic medical conditions, such as ischemic heart disease and diabetes mellitus as well as impaired interferon type I responses, are risk factors for developing more severe coronavirus disease 2019 (Covid-19) [11, 12]. Further, severe Covid-19 is frequently associated with increased inflammation, lymphopenia and occasionally a proinflammatory cytokine storm [9, 10, 12]. Since Covid-19 was a new disease entity, it was not known how patients with different inborn errors of immunity (IEI) would cope with the disease. Early reports of Covid-19 in patients with XLA showed a milder disease progression than expected [13–15], but some reports showed severe clinical outcomes, where patients required hospitalization with intensive care [16, 17]. Given the role for hyperinflammation in severe Covid-19, it was suggested that absence of B cells may reduce this risk [10, 13]. Consequently, BTK-inhibitors were tested against Covid-19 induced respiratory distress. Notably, BTK-inhibitors did not improve the clinical outcome, even though inflammatory cytokines in plasma were reduced [18]. Another aspect that may be advantageous for patients with XLA, is that they cannot produce autoantibodies against type I interferons, which have been associated with critical Covid-19 in elderly people [19]. On the other hand, antigen specific antibodies play an important role in neutralizing viruses and facilitating and initiating phagocytosis [20]. To date, most studies have assessed the risk for severe SARS-CoV-2 infection in patients with IEI as one entity and only included small cohorts of patients with XLA [21–26]. Thus, despite the fact that it is almost 5 years since the pandemic started, we still do not have detailed information on risk factors for moderate to severe Covid-19 in patients with XLA. To address this question, we designed a study with the aim of describing the spectrum of SARS-CoV-2 related outcomes in patients with XLA and to identify risk factors for hospitalization due to a more severe infection. Data from 17 centers in Europe and the US was collected and used for the analyses.

## Methods and Materials

### Setting and Participants

**Fig. 1:**
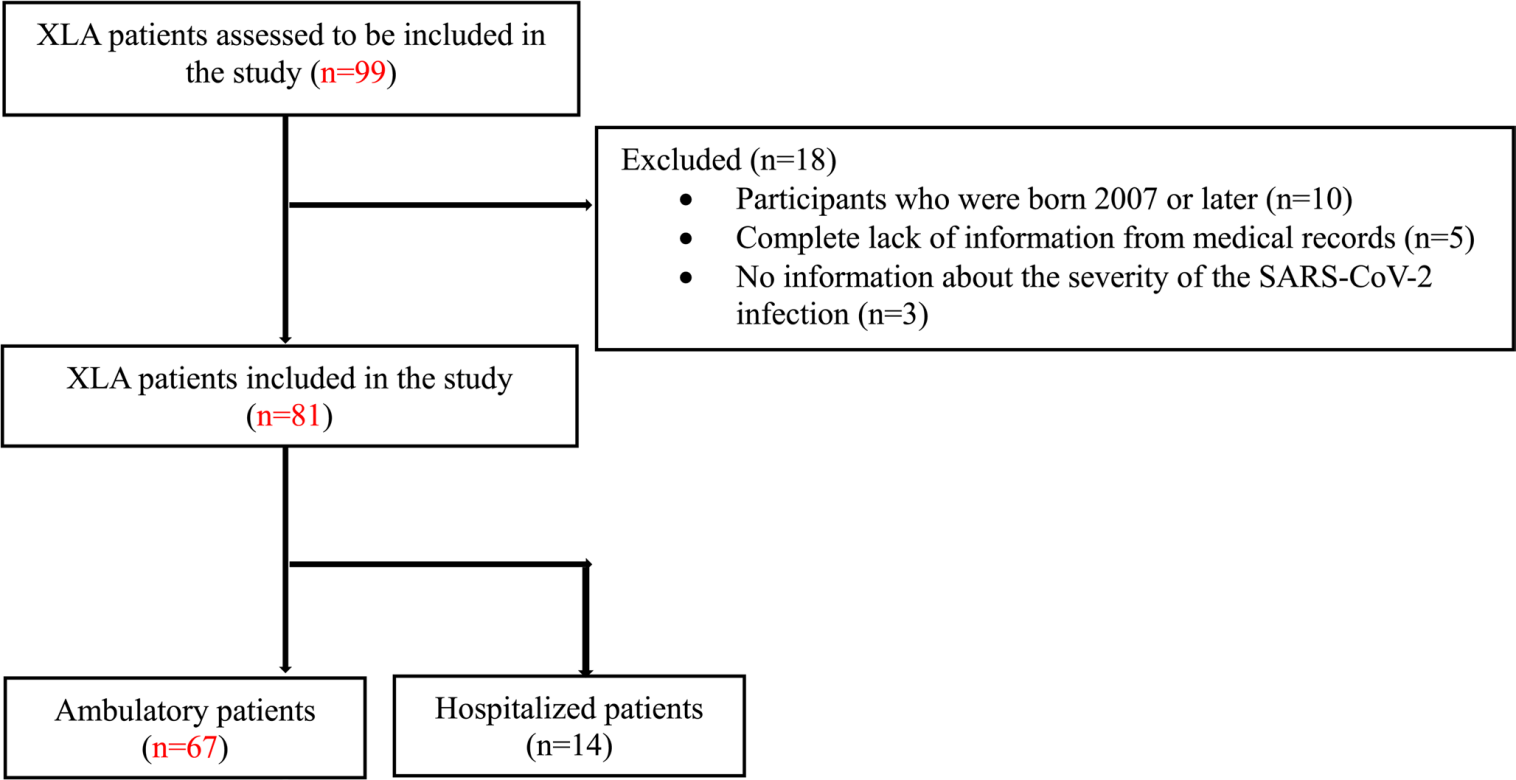
Flowchart of included XLA patients in the study

This is a retrospective, observational multicenter study following the RECORD statement guidelines and comprising 17 centers in Europe and the US during the SARS-CoV-2 pandemic. Data on 99 patients with XLA treated at these clinics have been collected until the beginning of year 2024. Patients over 18 years of age with an XLA diagnosis were included in the study. Participants without any data from medical records or without information on the severity level of the SARS-CoV-2 infection were excluded, see Fig. 1. A few patients have been described in separate published case reports.

The study period included the whole pandemic during the years 2020–2023. The primary investigators (PI) received a template with the pre-selected variables. Demographic and clinical variables were extracted from the patients’ medical records. Missing data was followed-up with further correspondence. The study participants were divided into two groups depending on the need for hospital care due to SARS-CoV-2 infection. The ambulatory group consisted of patients with either no diagnosed Covid-19 infection, asymptomatic infection, or mild ambulatory infection whereas the hospitalized group consisted of all patients with hospitalization because of their Covid-19 infection. Patients were diagnosed with SARS-CoV-2 infection with home antigen test or PCR test. All patients with a documented clinical severity degree of the Covid-19 infection were included, even when the PI had not specified the test method used (Suppl. Table 1). Six patients with no diagnosed Covid-19 infection were also included in the ambulatory group since testing for SARS-CoV-2 was not consistently available for patients with mild respiratory symptoms [10].

### Variables

The following variables were analyzed: the year of birth, biological sex, BTK protein expression, pretreatment IgG level, last sample of Ig levels including different isotypes (IgG, IgM and IgA) before Covid-19 infection, B cell counts, baseline T cell phenotype and counts including total CD4^+^ T cell counts, total CD8^+^ T cell counts, percentage naive CD4^+^ and CD8^+^ T cells and CD57^+^ CD8^+^ T cells (senescent/terminally differentiated cells) and TEMRA CD8^+^ T cells, baseline NK cell counts, secondary organ involvement due to primary antibody deficiency (PAD), degree of bronchiectasis (see below), impairment of lung function, medical risk factors, IgG trough values from Ig replacement therapy (IgRT) and the type of administration, current or recent immunosuppressive therapy before infection, lowest lymphocyte, neutrophil and monocyte counts during infection, vaccination status, SARS-CoV-2 antibody level, SARS-CoV-2 T cell response, date of infection and type of SARS-CoV-2 diagnostics.

Covid-19 specific symptoms were registered as well as severity of disease with organ involvement, hospitalization, superinfections, Covid-19 specific treatment and outcome. Covid-19 specific therapy was categorized as antiviral or anti-inflammatory therapy.

The severity level of bronchiectasis was graded from 0 to 3 based on imaging results. Absence of bronchiectasis was graded as 0, mild disease with few small bronchiectasis was graded as 1, intermediate disease with prominent bronchiectasis in 1–2 lobes was graded as 2 and severe bronchiectasis in *≥* 2 lobes was graded as 3. Impairment of lung function was ranked from spirometry values as the following: 0 = normal function, 1 = mild obstructive lung disease or restrictive lung disease and 2 = intermediate to severe obstructive or restrictive lung disease. The following medical risk factors for hospitalization during SARS-CoV-2 infection based on the literature [11, 27] were reported: diabetes, hypertension, cardiac disease, chronic lung disease, cerebrovascular disease, chronic kidney disease, cancer, smoking, or other medical risk factors specified by the primary investigator. The other included medical risk factors were post lung transplantation and obesity (BMI ≥ 30 [28]). Risk factors specified by the primary investigator without significant evidence from the literature were not part of the analysis, which included epilepsy, cognitive impairment following viral encephalitis, overweight (BMI ≥ 25 and < 30) and chronic sinusitis. The patients were grouped into no known medical risk factor, one medical risk factor, or *≥* 2 medical risk factors. The vaccination status included the types of vaccines, the number of doses before infection and the date of the last dose. The SARS-CoV-2 antibody levels were measured in binding antibody units (BAU)/ml or measured in U/ml with interpreted results. The anti-SARS-CoV-2 RBD S1 IgG cut-off for positive result was 50 BAU/ml based on a study comparing antibody levels after natural Covid-19 infection to the WHO International Standard and Reference Panel for anti-SARS-CoV-2 antibody [29, 30]. One of the included test results was presented in arbitrary units (AU)/ml from Abbott and the result was regenerated with the conversion factor from the company to calculate WHO BAU/ml [31, 32]. The SARS-CoV-2 specific T cell response after vaccination and before infection was categorized based on the level of the measured response, ranging from absent to poor, intermediate or strong response. The superinfections included bacterial or fungal pneumonia and sepsis. Outcome was categorized as 0 = completely restored to previous condition, 1 = mild sequelae, 2 = severe sequelae other than long Covid-19, 3 = long Covid-19 and 4 = death.

### Statistical Analysis

The analyses were performed in GraphPad Prism version 10.0.3 and R Studio 4.3.1. Since the study was hypothesis-generating, we chose an explorative design with multiple testing on all parameters. First, univariate analyses were performed in GraphPad on all relevant variables comparing the groups. Variables with a high proportion of missing data or descriptive data without possible intergroup comparisons were not analyzed. Categorical ordinal data was analyzed with Mann Whitney U-test and continuous data was analyzed using Welch’s t-test due to small sample size. Binominal data was analyzed with Fisher’s exact test. Simple logistic regression was used on all relevant variables with p value *< 0.05* in GraphPad. Due to small sample size, we chose to confirm our results and calculate more accurate confidence intervals with Firth’s Bias-Reduced Logistic Regression, using the logistf package in R studio. Thereafter, multiple logistic regression was performed with Firth’s Bias-Reduced Logistic Regression, in a stepwise selection model to study how the different variables correlated with risk for hospitalization.

During the regression analysis, the variables were converted into dichotomous variables based on the median values to only study risk for hospitalization. We studied risk for hospitalization during an early observation period (specified in the result section) and for the whole observation period. Since we wanted to study an earlier phase of the pandemic than before January 2022, we chose to divide the patients into infection up until November 2021 (early phase before Omicron was the prominent viral variant) or afterwards (late phase with Omicron variant).

Further, we divided the T cell population based on the lower 25th percentile of the normal range of CD4^+^ T cells counts; 617 cells/µl, and called the variable “low CD4^+^ T cell counts” [33].

### Standard Protocol Approvals

All medical centers have extracted data and have collected informed patient consent in compliance with local ethical regulations. An ethical approval to compile all data was approved by the Swedish Ethical Review Authority (dnr 2024–03237-01).

## Results

### Most of the Infections Requiring Hospitalization Occurred Early in the Pandemic

A total of 99 patients were screened for inclusion in the study. However, 18 patients were excluded due to young age (< 18 years) or lack of medical information (Fig. 1). All included study participants were biological males due to the X-linked inheritance pattern for the disease.

**Fig. 2:**
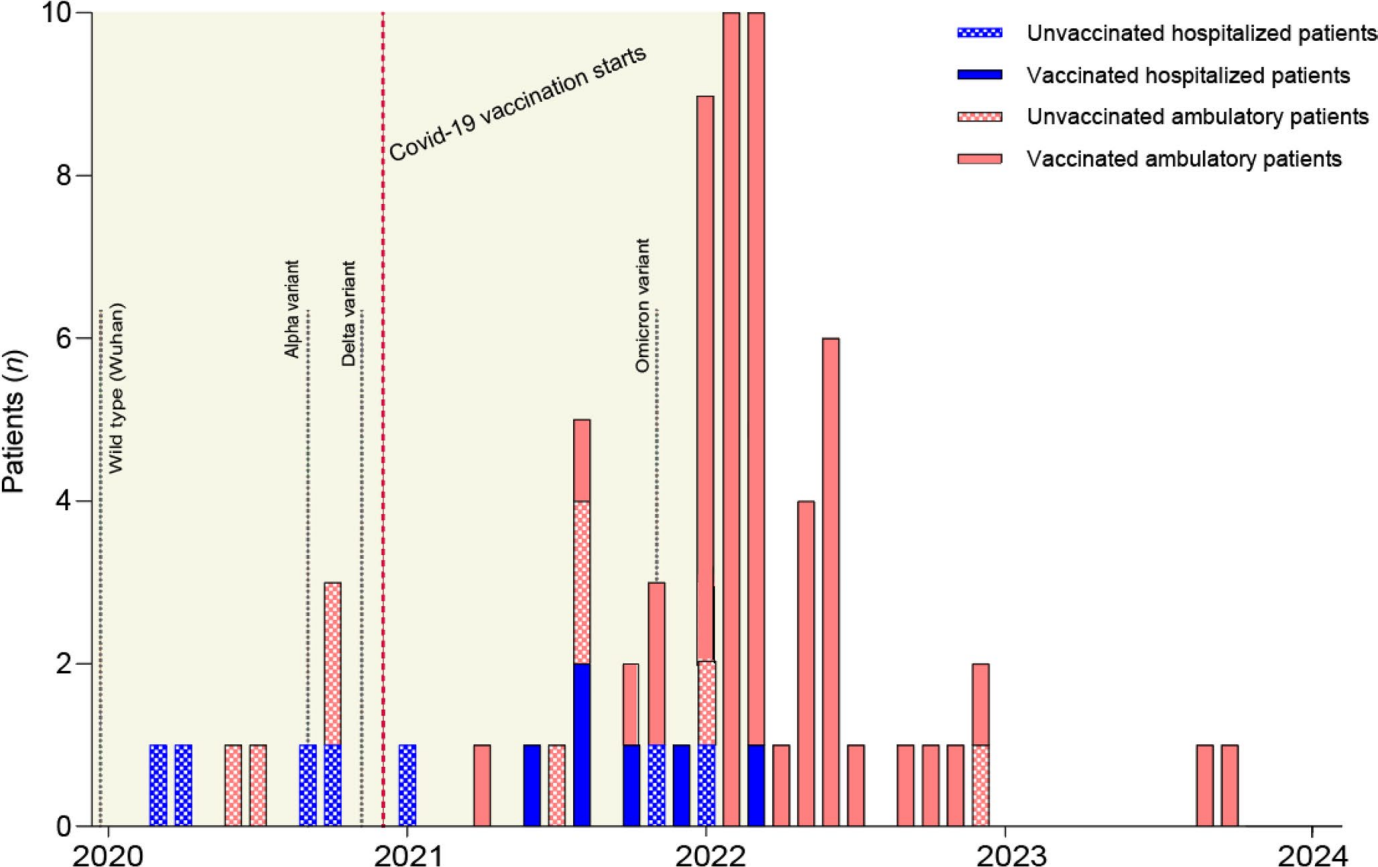
Timeline chart of SARS-CoV-2 infections in patients with XLA in relation to hospitalization and vaccination status. The date of the first infection was categorized as months after December 2019. The graph shows number of infected patients on the Y-axis and the first date of infection on the X-axis. The ambulatory (red) vs. hospitalized state (blue) due to the SARS-CoV-2 infection and the vaccination status before infection are indicated. The early observation period (2020–3/2022) is highlighted. On average, hospitalized infections occurred 9 months [95% CI 4–15] before ambulatory infections, *p* < 0.01 (Welch’s t test). A total of 71 patients are included in the figure, since 3 ambulatory patients and 1 hospitalized patient had no exact date of infection, and 6 patients had no known infection, and were therefore excluded. 1 ambulatory patient got infected during spring 2021 and was categorized with the mean value between March – May 2021 and 1 ambulatory patient got infected during 2020 and was categorized with the mean value between January-December 2020. The emergence of the most frequent SARS-CoV-2 variants of concern are plotted along the time line [34]

The pandemic had several phases with different circulating viral variants and vaccination schedules across Europe and in the US, which affected vaccination status and the presence of anti-SARS-CoV-2 specific antibodies in the IgRT preparations. Therefore, it was important to first assess the impact of calendar time on Covid-19 severity. Early infection date was correlated with hospitalization; all infections requiring hospitalization (14/81 = 17%) occurred up until March 2022 (Fig. 2). In addition, a large increase in milder infections was seen during the Omicron period, which necessitated compensation for the skewed distribution over time. Thus, we decided to focus our further investigations on the hospitalized patients versus the early ambulatory patients (41/81 = 51%) with an asymptomatic infection or mild ambulatory infection up until March 2022, to identify additional risk factors for hospitalization (Table 1). The late ambulatory patients (*n* = 26/67 = 39%), with an infection after March 2022 or without an overt infection, were hence excluded from the main analysis (Table 1). In the hospitalized group, only one patient received high flow oxygen therapy or non-invasive ventilation (NIV) and two patients needed mechanical ventilation (Suppl. Table 1). All patients with reported reinfections had mild ambulatory reinfections, and there was no significant difference in the numbers of reinfections between the groups (Suppl. Table 1). There was no significant difference between the mean age of the groups (31 years in the early ambulatory group versus 35 years in the hospitalized group, *p* = 0.21).

**Table 1:** Demographic, clinical and immunological parameters of XLA patients

	All participants *n* = 81	Early ambulatory group, *n* = 41	Hospitalized group *n* = 14	*p*-value	Late ambulatory group, *n* = 26
Age, mean (SD)^a^	34 (12)	31 (11)	35 (8)	*0.21*	42 (13)
<25 years, n (%)	18 (22)	15 (37)	1 (7)		2 (8)
25–50 years, n (%)	54 (67)	25 (61)	12 (86)		17 (65)
>50 years, n (%)	9 (11)	1 (2)	1 (7)		7 (27)
Date of infection in months after December 2019, mean (SD)^b^	24 (8)	23 (6)	17 (8)	***0.02***	33 (5)
Last IgG value (g/L) before SARS-CoV-2 infection, mean (SD)^c^	9.6 (2.3)	9.9 (2.2)	7.7 (1.8)	***< 0.01***	10.2 (2.2)
Vaccination before infection, *n (%)*	64 (79)	32 (78)	7 (50)	*0.09*	25 (96)
Number of vaccinations, median (range)^d^	2 (7)	2 (4)	1 (3)	*0.12*	4 (7)
Days between last vaccination and Covid-19 infection, mean (SD)^e^	134 (94)	111 (65)	64 (45)	*0.08*	196 (115)
Bronchiectasis disease, *n (%)*^f^	38 (48)	14 (34)	11 (85)	***< 0.01***	13 (50)
Bronchiectasis severity level, *n (%)*^g^				***< 0.01***	
Grading 0 (= absent)	42 (53)	27 (66)	2 (15)		13 (50)
Grading 1	19 (24)	7 (17)	6 (46)		6 (23)
Grading 2	15 (19)	7 (17)	3 (23)		5 (19)
Grading 3	4 (5)	0 (0)	2 (15)		2 (8)
Superinfection, n (%)^h^	14 (19)	3 (7)	8 (57)	***< 0.01***	3 (15)
Severity of superinfection, *n (%)*^i^				*0.08*	
Untreated	1 (7)	1 (33)	0 (0)		0 (0)
Mild (treated)	4 (28.5)	1 (33)	1 (12.5)		2 (67)
Intermediate	5 (36)	1 (33)	3 (37.5)		1 (33)
Severe	0 (0)	0 (0)	0 (0)		0 (0)
Critical	4 (28.5)	0 (0)	4 (50)		0 (0)
Superinfection in bronchiectasis patients, *n (%)*^j^	11 (32)	1 (7)	8 (73)	***< 0.01***	2 (22)
Total CD4^+^ counts (/µl), mean (SD)^k^	990 (527)	1054 (472)	709 (223)	***< 0.01***	1015 (661)
Total CD8^+^ counts (/µl), mean (SD)^k^	628 (365)	602 (364)	484 (256)	*0.29*	728 (392)
NK cell counts (/µl), mean (SD)^l^	183 (167)	221 (83)	111 (75)	***0.02***	165 (172)
Lowest lymphocyte count (/nL) during infection, mean (SD)^m^	1.4 (0.9)	1.7 (0.6)	0.6 (0.5)	***< 0.01***	1.8 (0.9)
Lowest monocyte count (/nL) during infection, mean (SD)^n^	0.6 (0.3)	0.7 (0.2)	0.4 (0.3)	***< 0.01***	0.7 (0.3)

P-values refer to the comparison between patients who did or did not require hospitalization for SARS-CoV-2 infection during the early phase of the observation period. All footnotes are shown below in Supplementary

^a^The year born was converted to the expected age the year of 2024. Welch’s t test was used to compare the mean age in the two groups

^b^Date of infection in months after December 2019. Analysis of 71 patients with results available. 3 of the early ambulatory patients had no specific date of infection, but were evaluated of outcome after Covid-19 up until March 2022. They were therefore part of the early ambulatory group but excluded from this analysis

^c^In total, 75 patients had results available. 49 patients were included in the Welch’s t test

^d^In total, 80 patients had results available. One vaccinated patient in the late ambulatory group had several vaccinations but the exact number of doses before infection was not known due to separate medical records. The patient was excluded from the analysis

^e^Analysis of 52 patients with results available. If the date was specified with month and year, the date of the 15^th^ was decided for the specific month. 7 patients in the ambulatory group had only month and year specified for the last vaccination date before infection compared to 2 patients in the hospitalized group. 18 patients in the ambulatory group had only month and year specified for the date of SARS-CoV-2 infection compared to 3 patients in the hospitalized group

^f^In total, 80 patients had results available. One patient in the ambulatory group had lung transplantation because of severe bronchiectasis before the SARS-CoV-2 infection and was converted to absence of bronchiectasis

^g^In total, 80 patients had results available. The analysis was performed with Mann-Whitney U test. One patient in the ambulatory group had bronchiectasis in the right middle lobe and was categorized as 1. One patient in the ambulatory group had basal cylindric bronchiectasis in left lobe and one high resolution computed tomography that mentioned bronchiectasis in lingula and was categorized as 1. One patient in the hospitalized group had computed tomography during severe Covid-19 infection and the bronchiectasis severity level of 1 was based on that diagnostic imaging. One patient in the hospitalized group was diagnosed with bronchiectasis but no grading was available from the medical records, and the mean group value of 1 was imputed for the patient

^h^The 6 patients in the late ambulatory group with no diagnosed Covid-19 infection were excluded from the statistics. One patient in the late ambulatory group had sinusitis specified as superinfection and was reported as no superinfection

^i^Analysis of the 11 patients who were reported with superinfection with Mann-Whitney U test

^j^In total, 34 patients with bronchiectasis were analyzed, since 4 bronchiectasis patients had no known Covid-19 infection and were excluded

^k^In total, 61 patients had results available. 40 patients were included in Welch’s t test

^l^In total, 48 patients had results available. 33 patients were included in the Welch’s t test

^m^In total, 30 patients had results available. 21 patients were included in the Welch’s t test

^n^In total, 29 patients had results available. 20 patients were included in the Welch’s t test

## Lower Levels of Total IgG from IgRT were Associated with an Increased Risk for Hospitalization

**Fig. 3:**
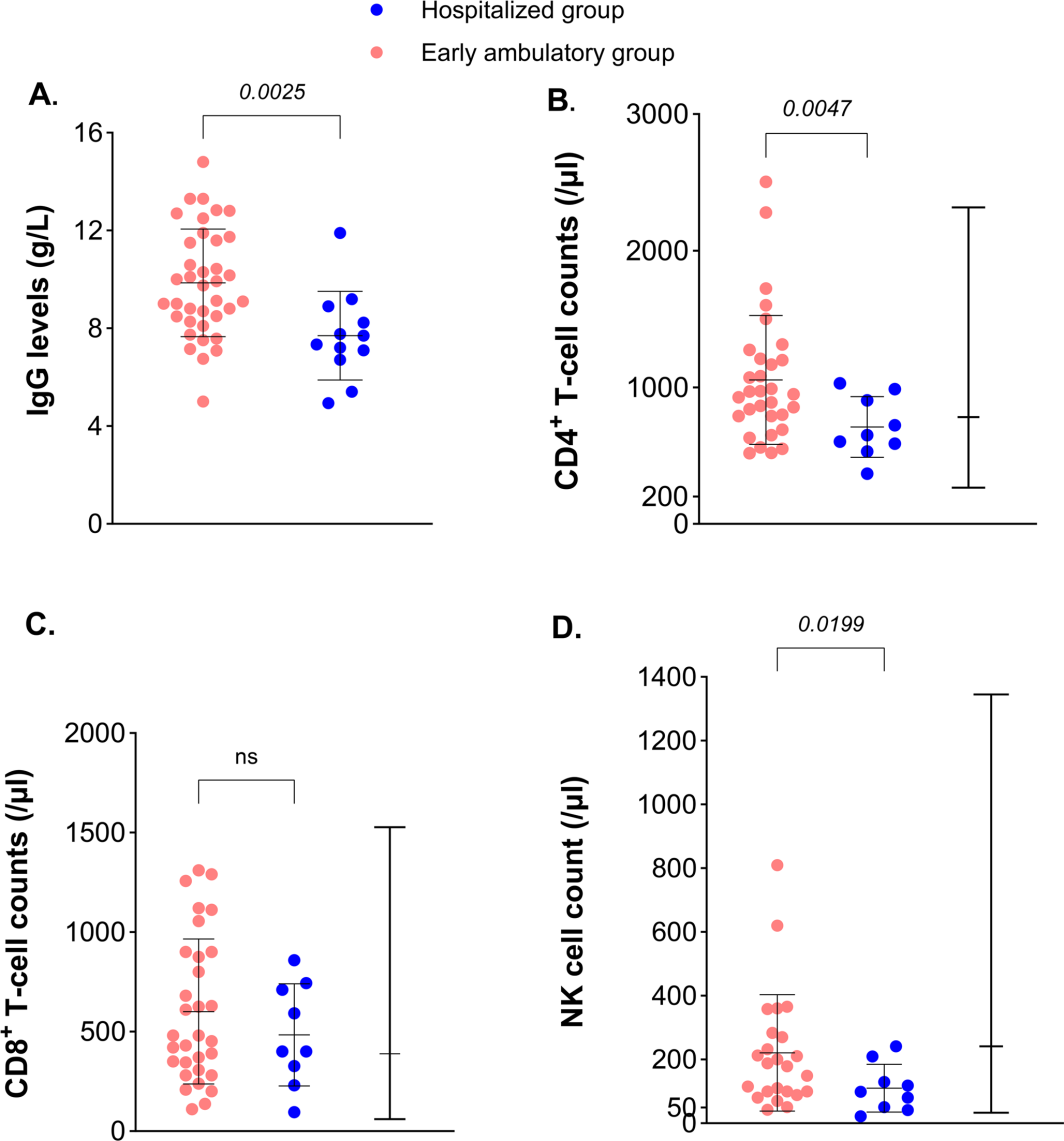
Pre-infection IgG trough levels and lymphocyte subpopulations in patients with XLA. (**A**). IgG levels (g/L) before SARS-CoV-2 infection comparing the early ambulatory patients (pink) with the hospitalized patients (blue). Welch’s t-test was performed. (**B**). Baseline CD4^+^ T cell counts comparing the groups with Welch’s t-test, the normal range with median is shown to the right [33]. (**C**). Baseline CD8^+^ T cell counts comparing the groups with Welch’s t-test, the normal range with median is shown to the right [33]. (**D**). Baseline NK cell counts comparing the groups with Welch’s t-test, the normal range with median is shown to the right [33]

**Fig. 4:**
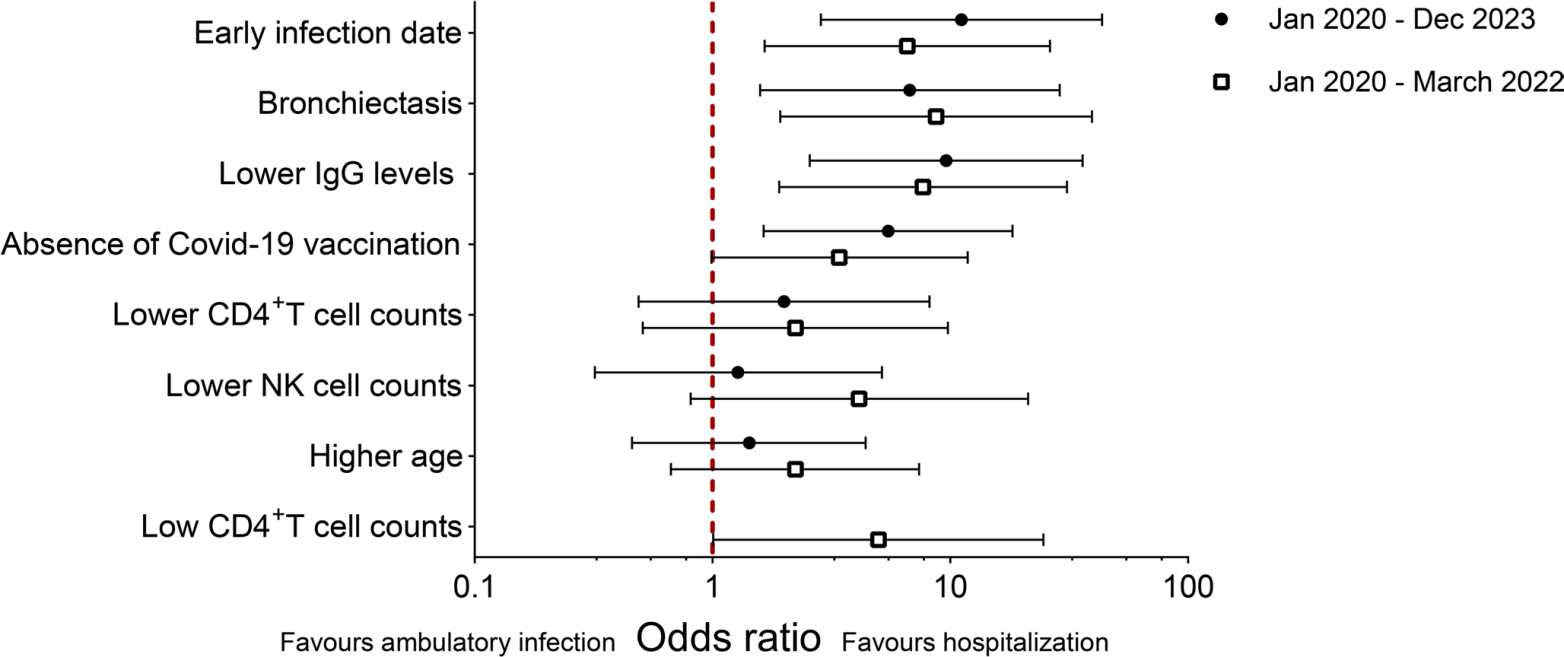
Risk factors for SARS-CoV-2 infection requiring hospitalization. Forrest plot of the univariate analysis on variables significantly associated with hospitalization of SARS-CoV-2 infection in XLA patients. All variables have been reanalyzed into dichotomous variables (under or median value for all study participants versus above median value for all study participants, or as specified in the statistical analysis) to only study risk for hospitalization

First, we assessed whether the IgG trough levels from IgRT differed between the groups. The level of 8 g/L has been suggested as a target to protect against infection in antibody deficient patients [35, 36]. Patients in the early ambulatory group had a higher mean IgG trough level (9.9 g/l) compared to the hospitalized group (7.7 g/l), *p* < 0.01 (Fig. 3A). Indeed, lower IgG levels (< 8 g/L) were independently associated with higher risk for hospitalization, but with broad confidence intervals (OR 7.68 [95% CI 1.91–30.97] in the univariate analysis), (Table 2; Fig. 4). This risk was also detected in the analysis on the whole cohort; lower IgG levels (< 8 g/L) were consistently associated with higher risk for hospitalization regardless of bronchiectasis, vaccination, age and date of infection (Suppl. Table 3, Fig. 4). The route of IgG administration did not differ between the hospitalized and non-hospitalized early group (*p* = 0.51, Suppl. Table 1).

**Table 2:** Logistic regression analysis of factors associated with hospitalization due to SARS-CoV-2 infection in patients with XLA in the early observation period

	Univariate analysis		Multivariable analysis^a^		Multivariable analysis^b^		Multivariable analysis^c^		Multivariable analysis^d^	
	OR (95% CI)	*p* value	OR (95% CI)	*p* value	OR (95% CI)	*p* value	OR (95% CI)	*p* value	OR (95% CI)	*p* value
Higher age	2.24 (0.67–7.41)	*0.19*								
Bronchiectasis	8.72 (1.93–39.43)	***< 0.01***	13.80 (1.91–99.72)	***< 0.01***	15.07 (2.66–85.44)	***< 0.01***	***–***		11.29 (2.05–62.18)	***< 0.01***
Lower CD4^+^ T cell counts	2.24 (0.51–9.79)	*0.28*								
Low CD4^+^ T cell counts^e^	5.00 (1.01–24.65)	***0.048***								
Lower NK cell counts	4.14 (0.81–21.29)	*0.09*								
Lower IgG levels	7.68 (1.91–30.97)	***< 0.01***	***–***		11.84 (2.16–64.82)	***< 0.01***	15.64 (2.36–103.6)	***< 0.01***	11.25 (1.74–72.79)	***0.01***
Early infection date	6.60 (1.66–26.29)	***< 0.01***	4.30 (0.92–20.15)	*0.06*	4.22 (0.88–20.25)	*0.07*	7.63 (1.58–36.84)	***0.01***	***–***	
No Covid-19 vaccination	3.42 (0.99–11.87)	*0.05*	6.94 (1.37–35.23)	***0.02***	***–***		9.50 (1.71–52.90)	***0.01***	2.06 (0.43–9.84)	*0.37*

The multivariable analyses are performed on all 55 patients (14 hospitalized patients and 41 ambulatory patients) with infection in the early observation period. 3 of the patients had no specified date of infection, but had a specified date of last evaluation of outcome after the Covid-19 infection before or under March 2022, and were therefore included in the analysis

^a^Odds ratio adjusted for the two covariates high age and low IgG levels before infection

^b^Odds ratio adjusted for the two covariates high age and no Covid-19 vaccination before infection

^c^Odds ratio adjusted for the two covariates high age and bronchiectasis

^d^Odds ratio adjusted for the two covariates high age and early date of infection

^e^The variable has been categorized based on the lower 25th percentile of the normal range of CD4^+^ T cells (above or below 617 cell/µL) [33],

The variables have been reanalyzed into dichotomous variables; under or median value for all study participants versus above median value for all study participants. Since the median value for infection date was January 2022, we chose to divide the groups after November 2021 to study the Omicron period until March 2022 versus the earlier variants of concern. Lower IgG levels were categorized as < 8 g/L as mentioned in the method

### Bronchiectasis was a Risk Factor for Hospitalization

Next, we set out to understand which clinical factors defined patients who required hospitalization due to moderate or severe disease. Notably, there was a significantly higher prevalence of bronchiectasis in the hospitalized group when compared to the early ambulatory group, even with milder cases of bronchiectasis (Table 1). 85% of hospitalized patients had bronchiectasis compared to 34% of the early ambulatory patients (*p* < 0.01). The presence of bronchiectasis correlated with an increased risk for hospitalization when adjustments were made for age, IgG levels, vaccination and date of infection during both observation periods, but with broad confidence intervals (Table 2 and Suppl. Table 3). We also found that both mild and severe bronchiectasis increased risk for hospitalization in the whole cohort (Suppl. Table 2). We found that superinfections were more common in the hospitalized group compared to the early ambulatory group (57% versus 7%, *p* < 0.01). The severity of superinfection was clearly different between the groups, but likely due to small sample size, no significant p-value was found (*p* = 0.08), (Table 1). In the hospitalized group, four patients had bacterial pneumonia, three patients had fungal pneumonia, and one patient had bacterial and fungal pneumonia with invasive fungal infection. In the whole ambulatory group, all six patients with superinfections had bacterial pneumonia and no fungal infections or sepsis were reported.

Further, a higher proportion of superinfections was observed in hospitalized patients with bronchiectasis compared to early ambulatory patients with bronchiectasis (73% versus 7%, *p* < 0.01), (Table 1). When testing how superinfection was correlated to bronchiectasis itself, we found that 9/25 = 36% of patients with bronchiectasis developed a superinfection during Covid-19, compared to only 2/29 = 7% of patients without bronchiectasis (*p =* 0.02).

Interestingly, lung function did not differ between the groups (*p* = 0.31) (Suppl. Table 1). Furthermore, no significant differences between the groups were shown regarding secondary organ involvement, signs of immune dysregulation or for known risk factors connected to severe Covid-19 (*p = > 0.99* resp. *p* = 0.40), (Suppl. Table 1). Also, no differences were observed for concurrent immunosuppression (5% in the early ambulatory group had immunosuppressive treatment versus 0% in the hospitalized group, *p = > 0.99*).

In conclusion, bronchiectasis and superinfections were associated with Covid-19 hospitalization.

### Higher Levels of T- and NK-cells at Baseline were Found in the Non-hospitalized Group

Next, we analyzed the immunological profile of patients before SARS-CoV-2 infection (baseline). Patients with residual B cell counts ≥ 5/µl accounted for 6% of the early ambulatory group and 0% of the hospitalized group (*p* > 0.99), (Suppl. Table 1). No significant difference was found between the groups regarding detectable IgM or IgA levels measured before the Covid-19 infection (17% of the ambulatory group versus 0% of the hospitalized group, *p* = 0.32, and 11% of the early ambulatory group versus 8% of the hospitalized group, *p* > 0.99, respectively), (Suppl. Table 1).

The early ambulatory group had higher – and even increased - counts of total CD4^+^ T cells at baseline compared to the hospitalized patients (1054/µl versus 709/µl, *p* < 0.01) (Table 1; Fig. 3B). Likewise, the early ambulatory group displayed a higher mean NK cell count at baseline (221/µl versus 111/µl, *p* = 0.02), (Table 1; Fig. 3D). However, no association with risk for hospitalization was observed in the regression analysis for CD4^+^ T cell counts, or NK cell counts, due to high median values in the cohort (Table 2 and Suppl. Table 3, Fig. 4). Therefore, we reanalyzed the data with the cut-off from the lower 25th percentile of the normal range of CD4^+^ T cells [33], and our findings suggested a protective effect of CD4^+^ T cells above 617 cells/µl, but with broad confidence intervals. Based on the limited information collected, we could not identify significant differences regarding other T cell subpopulations (Suppl. Table 1). During infection, patients that required hospitalization exhibited lower counts of both lymphocytes and monocytes compared to the early ambulatory group (mean value of 1.7/nL for lymphocytes in the early ambulatory group versus 0.6/nL in the hospitalized group, *p* < 0.01, and mean value of 0.7/nL monocytes in the early ambulatory group versus 0.4/nL in the hospitalized group, *p* < 0.01, respectively). However, the hospitalized group received glucocorticosteroids to a larger extent, which may have affected the results (Table 3).

**Table 3:** Comparing the SARS-CoV-2 specific form of treatment between the early ambulatory and hospitalized groups

	Early ambulatory group *n* = 39^*a*^	Hospitalized group *n* = 14	*p*-value	Late ambulatory group *n* = 20^*a*^
Antiviral treatment, *n (%)*				
No treatment	28 (72)	2 (14)	***<0.01***	8 (40)
Casirivimab/imdevimab	3 (8)	4 (29)		1 (5)
Sotrovimab	5 (13)	3 (21)		2 (10)
Convalescent plasma	0 (0)	4^c^ (29)		0 (0)
Pentaglobin™	0 (0)	1 (7)		0 (0)
Remdesivir	1 (3)	5 (36)		0 (0)
Nirmatrelvir/Ritonavir	2 (5)	1 (7)		8 (40)
Molnupiravir	2 (5)	0 (0)		2 (10)
Anti-inflammatory treatment, *n (%)*				
No treatment	38 (97)	6 (43)	***< 0.01***	0 (0)
Prednisolone	1^b^ (3)	4 (29)		0 (0)
Dexamethasone	0 (0)	5 (36)		0 (0)
Tocilizumab	0 (0)	2 (14)		0 (0)
Anakinra	0 (0)	1 (7)		0 (0)

^a^In the early ambulatory group, 2 patients with missing data were excluded from the analysis. In the late ambulatory group, 6 patients with no known infection were excluded from the analysis

^b^Methylprednisolone was given due to SARS-CoV-2 infection during acute graft rejection post lung transplantation

^c^One patient was PCR positive for 270 days and received the treatment day 210

### Vaccination was Associated with Protection against Hospitalization during the Whole Period

Fewer of the hospitalized patients were vaccinated before infection, compared to patients in the ambulatory group (50% versus 78%, *p* = 0.09). Most patients received mRNA vaccines (29/32 in the ambulatory group; all in the hospitalized group). The absence of Covid-19 vaccination did not reach statistical significance in the unadjusted univariate analysis (OR 3.49, 95% CI 0.99–11.87, Table 2; Fig. 4). However, after adjusting for age, bronchiectasis and IgG levels in the multivariable analysis, absence of Covid-19 vaccination reached statistical significance for an increased risk of hospitalization (OR 6.94, 95% CI 1.37–35.23) (Table 2). Similarly, during the whole observation period, absence of Covid-19 vaccination was associated with a significantly increased risk of hospitalization, after adjustment for age, bronchiectasis and IgG levels (OR 5.48, 95% CI 1.64–18.31, Suppl. Table 3).

SARS-CoV-2 specific T cell responses after vaccination and before infection were assessed in 7 of the ambulatory patients for whom data was available. A normal to strong response after antigen-specific stimulation of memory T cells was demonstrated for six patients. Thus, SARS-CoV-2 vaccination seemed to be associated with a diminished risk for hospitalization due to moderate to severe Covid-19 in patients with XLA when the whole observation period was evaluated.

### Hospitalized Patients Developed More Severe Covid-19 Symptoms

As expected, the symptom burden was larger in the hospitalized group compared to the ambulatory (A) group; 71% of hospitalized patients developed fever (vs. 28% in the A-group), 21% had respiratory symptoms (vs. 7% in the A-group) and 29% of the patients had dyspnea at rest (vs. 1% in the A-group). In addition, 7% of hospitalized patients had symptoms of the central nervous system (vs. 0% in the A-group).

Organ involvement during Covid-19 also differed between the groups. In the hospitalized group, 93% of the patients had lung involvement (vs. 18% in the A-group), 14% had renal involvement, 7% developed thrombocytopenia and neutropenia (vs. 0% in the A-group).

The SARS-CoV-2 specific antiviral treatment and anti-inflammatory treatment during infection differed between the groups. Notably, 86% of hospitalized patients received antiviral treatment, whereas only 28% in the early ambulatory group received specific antiviral treatment (*p* < 0.01), (Table 3).

## Discussion

We explored risk factors for Covid-19-related hospitalization in a large international cohort of patients with XLA. 17% of the enrolled patients required hospitalization due to Covid-19, but only 3 patients needed high flow oxygen or mechanically assisted ventilation.

In order to estimate the risk of hospitalization in patients with XLA, it is relevant to compare the Covid-19 hospitalization rate of XLA patients to those with CVID, who also have dysfunctional humoral immune responses with low to non-existing antibody production to neoantigens, and demonstrate various degrees of impairment in their B-cell lineage, however frequently combined with some kind of T-cell deficiency [37–39]. In our previous study, Covid-19 hospitalization occurred in 20% (34/168) of patients with CVID until January 2023 [40]. This resembles additional studies which have shown Covid-19 hospitalization rate in patients with CVID of 22% until April 2021, and of 12.6% until September 2022 [41, 42]. Another study found that 31% (4/13) of patients with CVID were hospitalized during the pandemic and that low IgG trough and lymphocyte counts were risk-factors [43]. A nationwide study have also shown increased risk for both hospital contact and hospitalization with severe Covid-19 for CVID patients compared to the general population [incidence risk ratio of 24.5 and 16.2 respectively] [44]. Thus, in our study the risk estimate for Covid-19 hospitalization for patients with XLA appears to be in the same range as for CVID patients, but this needs to be investigated further in studies comparing the groups directly.

All hospitalizations occurred during the early phase of the SARS-CoV-2 pandemic before March 2022. The relatively mild Covid-19 infections thereafter are probably due to the occurrence of omicron variant, increasing titers of SARS-CoV-2 specific antibodies in the plasma products for IgRT and the availability of vaccination. Omicron led to higher rates of breakthrough infections after vaccination, but with milder disease courses and reduced risk for hospitalization than during the Delta period [45]. This may be due to a slightly altered host-cell entry favoring upper respiratory epithelia over lung parenchyma compared to other previous variants of concern [46]. In regard to SARS-CoV-2 specific antibodies in the IgG preparations, we have shown that fewer than half of the IgRT batches produced after the start of the Covid-19 pandemic had a seropositive result, many with results near baseline [47]. Some patients therefore received convalescent plasma during hospitalization early in the pandemic, which may have helped the clinical recovery [48, 49]. The amount of anti-SARS-CoV-2 antibodies in IgRT batches increased over time during the pandemic as a reflection of the vaccination status in the donor population [47, 50–54]. Mostly, products manufactured in August 2021 or later had antibody titers similar to values in healthy vaccinated donors, due to the time-lapse of approximately 8 months from plasma donation to final commercial Ig products [51]. Further, the study showed that patients with primary antibody deficiencies (PAD) who were given Ig batches manufactured approximately 7.7 months (range 3–25 months) before infusion, had protective IgG levels in December 2021 and onwards [51]. Notably, the neutralizing capacity of the spike antibodies was consistently lower against the SARS-CoV-2 Omicron variants of concern emerging later in the pandemic [47, 50, 51]. Further, therapeutic monoclonal antibodies also showed limited neutralizing capacity against new circulating Omicron subvariants [55, 56]. All these factors most likely contributed to the strong influence of the time of infection on the risk of hospitalization, which hampers the identification of patient specific risk factors.

Therefore, we decided to focus on the first phase until March 2022 when the last XLA patient in our cohort was hospitalized. Focusing on this early observation period, we identified bronchiectasis (mostly complicated with superinfection) as well as IgG trough levels < 8 g/L as the main risk factors for hospitalization. It is known for other infections that higher IgG in patients with IEI and hypogammaglobinemia are associated with lower risk for infections and pneumonia [57–59]. The situation for protection against SARS-CoV-2 differs, however, from previous studies, since most IgRT preparations did not contain measurable or protective amounts of spike-specific IgG in the beginning of the pandemic [60]. Thus, higher IgG serum levels at this period of the pandemic most likely defended the patients against bacterial superinfections. However, we could not demonstrate a significant correlation between IgRT < 8 g/L and superinfection as outcome in our statistical analysis (data not shown). Superinfection was, however, clearly associated with the presence of bronchiectasis.

Bronchiectasis is common in patients with XLA; with approximately 60% of XLA patients over 30 years having developed bronchiectasis [7]. It was more common and more severe in the hospitalized group compared to the early ambulatory group. In a population registry study, it was shown that the presence of bronchiectasis increased the risk for moderate to severe SARS-CoV-2 infection compared to controls without bronchiectasis [61]. An increased risk has also been reported for other viral infections in antibody deficient patients with bronchiectasis [6], and influenza virus infections often cause hospitalization in patients with bronchiectasis [62]. However, bronchiectasis may have been a risk factor for Covid-19 hospitalization due to superinfection rather than due to Covid-19 itself, as there was a significantly increased risk of superinfection in patients with bronchiectasis. This would be in line with the observed protection by higher IgG levels as discussed above and previous knowledge that patients with XLA do not have a general susceptibility to severe viral infections, except invasive enteroviral infections [63, 64].

Cellular immunity in patients with XLA is also important, and we found that pre-infection levels of CD4^+^ T cells were significantly higher and even more frequently supranormal in the early ambulatory group compared to the hospitalized group. In our regression analysis, the data indicated protection of CD4^+^ T cells above 617 cell/µl, the lower 25th percentile of the normal range of CD4^+^ T cells [33]. This finding further supports a key role for an elevated amount of helper T cells to compensate for the loss of humoral immunity and protect against severe infection after vaccination. It has been shown that immunodeficient patients with pre-existing CD4^+^ T cells that cross-react with SARS-CoV-2, will have an increased response with activation of CD4^+^ memory T-lymphocytes directly after Covid-19 vaccination [65]. Notably, the hospitalized group still had CD4^+^ levels within the normal range [33], so hospitalization in the observed cohort was not due to an unexplained quantitative secondary cellular immunodeficiency. However, we cannot exclude differences in the previously reported alterations in the differentiation of T cells in patients with XLA [66, 67].

The same was true for baseline NK cell counts. NK cells are part of the innate immune system and may exert direct cytotoxic effector functions to cells invaded by viral pathogens [68]. Higher NK cell blood counts are correlated with a more rapid decrease in viral load during infections. It has been shown that peripheral blood NK cells decrease during SARS-CoV-2 infection while showing an activated phenotype [69]. However, the role of baseline NK cell counts in blood before SARS-CoV-2 infection remains unclear and further studies are needed.

Vaccination against SARS-CoV-2 has been described to decrease the risk of hospitalization due to Covid-19 in patients with IEI [70]. In our study, vaccination was also a protective factor when studying the effect during the whole observation period. A higher proportion of all ambulatory patients were vaccinated and had received a higher number of vaccine doses compared to the hospitalized patients. In a prospective clinical trial of the efficacy of the mRNA BNT162b2 vaccine, it was shown that 4 included patients with XLA did not exhibit any anti-SARS-CoV-2 spike antibodies 14 days after the second vaccine dose, which was expected [71]. However, and more importantly, XLA patients produce functional memory T cells after Covid-19 vaccination, as measured by IFN- γ release assays [72]. A more detailed analysis of T cells in patients with XLA revealed that they had a strong response after vaccination with persistent and highly functional oligo/-polyclonal CD4^+^ and CD8^+^ memory T cells equal to healthy controls [65]. In our cohort, 6 out of 7 tested participants showed a T cell mediated response after Covid-19 vaccination when stimulated with SARS-CoV-2 antigen in vitro. Thus, XLA patients have a normal T cell response to vaccination, which potentially contributes to the protection against severe infection. Definitive conclusions about the overall effect of vaccination on hospitalization remain, however, difficult due to the overlapping effects of passive immunization by IgRT.

The strength of this study is the large cohort of patients with XLA with different geographical origin. The relatively large dataset enabled adjustment for different confounders. After matching for age, concomitant diseases and date of infection, the collected data could generate statistically significant intergroup comparisons on clinical characteristics, medical therapies and immunological phenotypes.

There are also important limitations that need to be taken into account. Data extraction was done by separate primary investigators without any specific scoring systems. Thus, there may have been a discrepancy in categorizing the patients regarding lung impairment, the severity of bronchiectasis, medical risk factors, Covid-19 specific symptoms, organ involvement during Covid-19, superinfection severity degree and outcome. Further, there was missing data on several variables, which reduced the statistical power and may potentially have introduced bias. Only a few patients had results from extended baseline T cell phenotyping, BTK protein expression and SARS-CoV-2 specific antibodies before infection. Further, we did not study differences in when Covid-19 specific antiviral therapy was given in the two groups due to missing data.

## Conclusion

To summarize, this study shows that most hospitalizations due to Covid-19 in patients with XLA occurred early in the pandemic. The presence of bronchiectasis stood out as a prominent risk factor for hospitalized Covid-19 with subsequent superinfection. The main protective factors were high IgG trough levels possibly by protecting against superinfection, and possibly Covid-19 vaccination. We believe that these results suggest the critical role of an optimal IgG replacement therapy reaching trough levels above 8 g/L, especially in patients with bronchiectasis to protect patients with XLA during novel viral infections and reduce morbidity due to superinfections.

## Supplementary Information

Below is the link to the electronic supplementary material.

Supplementary file1 (DOCX 40.6 KB)

## Data Availability

No datasets were generated or analysed during the current study.
