# Supplementary material for: Bronchiectasis, Low IgG Levels and Lack of Vaccination are Risk Factors for Covid-19 Hospitalization in X-linked Agammaglobulinemia – A Retrospective Multicenter Study
Source: J Clin Immunol. 2025 Nov 15;45(1):161. doi: 10.1007/s10875-025-01962-3 (PMC12619783; doi:10.1007/s10875-025-01962-3)
Supplement: Supplementary file 1 — Supplementary file1 (DOCX 40.6 KB) [file 10875_2025_1962_MOESM1_ESM.docx]

**Supplementary**

**Supplementary Table 1** Clinical and immunological characteristics of XLA patients in the ambulatory and hospitalized group

| SARS-CoV-2 infection | All participants  *n=81* | | Early ambulatory group, *n=41* | Hospitalized group  *n=14* | Late ambulatory group, *n=26* | *p-value* |
| --- | --- | --- | --- | --- | --- | --- |
| Clinical severity level, n (%)^a^  No diagnosed infection  Asymptomatic infection  Mild symptomatic ambulatory disease  Hospitalized moderate disease without oxygen therapy  Oxygen therapy by mask or nasal prongs  Hospitalized severe disease with high flow oxygen therapy or NIV  Mechanical ventilation | | 6 (7)  5 (6)  56 (69)  4 (5)  7 (9)  1 (1)  2 (2) | 0 (0)  3 (7)  38 (93)  0 (0)  0 (0)  0 (0)  0 (0) | 0 (0)  0 (0)  0 (0)  4 (29)  7 (50)  1 (7)  2 (14) | 6 (23)  2 (8)  18 (69)  0 (0)  0 (0)  0 (0)  0 (0) |  |
| Outcome, *n (%)*  No complications  Mild sequelae  Severe sequelae  Long COVID  Death | | 75 (93)  2 (2)  1 (1)  2 (2)  1 (1) | 40 (98)  0 (0)  0 (0)  1 (2)  0 (0) | 9 (64)  2 (14)  1 (7)  1 (7)  1 (7) | 26 (100)  0 (0)  0 (0)  0 (0)  0 (0) | ***<0.01*** |
| Clinical characteristics | |  |  |  |  |  |
| Impairment of lung function, *n (%)*^b^  Grading 0  Grading 1  Grading 2 | | 53 (73)  6 (8)  14 (19) | 26 (74)  4 (11)  5 (14) | 8 (67)  0 (0)  4 (33) | 19 (73)  2 (8)  5 (19) | *0.31* |
| Secondary organ involvement due to PAD, *n (%)*^c^ | | 32 (41) | 12 (30) | 4 (33) | 16 (62) | *>0.99* |
| Medical risk factors, *n (%)*^d^  None  One  Two or more | | 39 (51)  30 (39)  7 (9) | 22 (55)  15 (38)  3 (8) | 6 (43)  6 (43)  2 (14) | 11 (50)  9 (41)  2 (9) | *0.40* |
| Ig replacement therapy at the time of Covid-19 infection, *n (%)*^e^  Subcutaneous injections  Intravenous infusion  No IgRT at infection start | | 50 (66)  25 (33)  1 (1) | 24 (63)  14 (37)  0 | 9 (69)  3 (23)  1 (8) | 17 (68)  8 (32)  0 (0) | *0.51* |
| Immunosuppression, *n (%)*^f^ | | 4 (5) | 2 (5) | 0 (0) | 2 (8) | ***>****0.99* |
| Humoral immunity | |  |  |  |  |  |
| Detectable B cells (>5/uL), n (%)^g^ | | 3 (4) | 2 (6) | 0 (0) | 1 (5) | *>0.99* |
| Pretreatment IgG level (g/l), mean (SD)^h^ | | 0.71 (1.35) | 0.54 (0.97) | 0.77 (1.83) | 0.96 (1.81) | *0.77* |
| Detectable IgM value (>0.1 g/L) before SARS-CoV-2 infection, n (%)^i^ | | 11 (15) | 6 (17) | 0 (0) | 5 (20) | *0.32* |
| Detectable IgA value (>0.07 g/L) before SARS-CoV-2 infection, n (%)^j^ | | 8 (11) | 4 (11) | 1 (8) | 3 (13) | *>0.99* |
| Cellular immunity | |  |  |  |  |  |
| Naive CD4^+^ (% of CD4^+^), mean (SD)^k^ | | 50.2 (15.1) | 51.2 (16.6) | 51.9 (11.7) | 48.4 (15.4) | *0.93* |
| Naive CD8^+^ (% of CD8^+^), mean (SD)^l^ | | 36.5 (25.9) | 44.4 (26.7) | 38.9 (10.7) | 25.7 (27.9) | *0.58* |
| CD57^+^ CD8^+^ (% of CD8^+^), mean (SD)^m^ | | 33.1 (21.07) | 23.5 (0.78) | 17.7 (16.9) | 49.8 (20.9) | *0.71* |
| TEMRA CD8^+^ (% of CD8^+^), mean (SD)^n^ | | 28.1 (18.3) | 21.4 (15.5) | 20.1 (11.1) | 35.2 (20.4) | *0.89* |
| Lowest neutrophil count (/nL)  during infection, mean (SD)^o^ | | 4.0 (2.0) | 4.0 (1.7) | 3.1 (2.3) | 4.9 (1.8) | *0.34* |
| Reinfection | |  |  |  |  |  |
| Reinfection with Covid-19, n (%)^p^ | | 19 (34) | 10 (30) | 5 (63) | 4 (27) | *0.12* |

***^a^****One patient with mild symptomatic ambulatory disease with Covid-19 specific symptoms had no diagnostics taken during the suspected infection due to lack of available tests early in the pandemic. Further, there were no information of the test method used regarding 2 of the patients with asymptomatic infections and 5 of the patients with mild ambulatory infections. One patient was hospitalized during SARS-CoV-2 infection for 10 days without further specification of the clinical severity level. The mean value of 4 for the hospitalized group was imputed for the patient.*

*^b^In total, 73 patients had results available. Mann-Whitney U test was performed on 47 patients in the hospitalized and early ambulatory group. Most patients had lung impairment classification done by the primary investigator. When the results were presented, the lung impairment classification was done according to Interpretative strategies for lung function tests (based on Pellegrino et al). One patient in the early ambulatory group had severe obstructive lung disease but had a lung transplantation before the SARS-CoV-2 infection. The actual lung impairment before infection was not known and the patient was excluded from the analysis. One patient in the early ambulatory group had no specified lung impairment other than light lung impairment and was categorized as 1. One patient in the early ambulatory group had FEV1 of 63 % and was categorized as intermediate obstructive lung disease. Another patient in the early ambulatory group had asthma and FEV1 of 83 %, FEV1/FVC of 0.95 and MEF25 of 65 % and was categorized as mild restrictive or obstructive lung disease. One patient in the late ambulatory group had FEV1/FVC of 0.66 and FEV1 of 68 % after bronchodilators and was categorized as intermediate obstructive lung disease. One patient in the early ambulatory group had impairment of lung function without any specification and was excluded from the analysis. One patient in the early ambulatory group had mild decrease of DLCOc/VA and normal ventilation capacity. The lung impairment was not possible to classify according to the guidelines, and the patient was excluded from the analysis.*

*^c^In total, 78 patients had results available. All other secondary organ involvement except lung involvement due to PAD were included in the results.*

*^d^In total, 76 patients had results available. The included medical risk factors are specified in the method section. Mann-Whitney U-test was performed.*

*^e^In total, 76 patients had results available. Fisher’s exact test was performed analyzing the difference in proportion of subcutaneous versus intravenous infusions in the two groups.*

*^f^In total, 80 patients had results available. One patient in the hospitalized group was treated with hydrocortisone due to adrenocortical insufficiency and was converted to no immunosuppression.*

*^g^In total, 67 patients had results available. Results < 5/uL were converted to 0. Fisher’s exact test was performed.*

*^h^In total, 45 patients had results available. 2 patients in the ambulatory group had Ig levels taken while breastfeeding, and for 1 patient in the ambulatory group, the PI was not completely sure if IgRT could had started. All results under detection level (ranging from < 0.33 g/l to <0.07 g/l) were converted to 0.*

*^i^In total, 72 patients had results available. Results under detection level for the specific lab were converted to 0.*

*^j^In total, 71 patients had results available. Results under detection level for the specific lab were converted to 0.*

*^k^In total, 29 patients had results available.*

*^l^In total, 24 patients had results available.*

*^m^In total, 7 patients had results available.*

*^n^In total, 18 patients had results available.*

*^o^In total, 31 patients had results available.*

*^p^In total, 56 patients had results available.*

**Reference:**

**Pellegrino R, Viegi G, Brusasco V, Crapo RO, Burgos F, Casaburi R, et al. Interpretative strategies for lung function tests. Eur Respir J. 2005 Nov;26(5):948-68.**

**Supplementary Table 2** **Logistic regression analysis of the impact of the severity of bronchiectasis on the risk for hospitalization in the whole observation period**

|  | Multivariable analysis^a^  OR (95% CI) | *p* value |
| --- | --- | --- |
| Bronchiectasis grading 1 | 9.48 (1.91 – 70.83) | ***0.01*** |
| Bronchiectasis grading 2 | 5.19 (0.77 – 43.45) | *0.09* |
| Bronchiectasis grading 3 | 24.46 (1.94 – 393.80) | ***0.01*** |

^a^Adjusted for age. The analysis was done with multiple logistic regression in GraphPad 10.0.3.

**Explanation of Foot notes to Table 1**

*^a^The year born was converted to the expected age the year of 2024. Welch’s t test was used to compare the mean age in the two groups.*

*^b^Date of infection in months after December 2019. Analysis of 71 patients with results available. 3 of the early ambulatory patients had no specific date of infection, but were evaluated of outcome after Covid-19 up until March 2022. They were therefore part of the early ambulatory group but excluded from this analysis.*

*^c^In total, 75 patients had results available. 49 patients were included in the Welch’s t test.*

*^d^In total, 80 patients had results available. One vaccinated patient in the late ambulatory group had several vaccinations but the exact number of doses before infection was not known due to separate medical records. The patient was excluded from the analysis.*

*^e^Analysis of 52 patients with results available. If the date was specified with month and year, the date of the 15^th^ was decided for the specific month. 7 patients in the ambulatory group had only month and year specified for the last vaccination date before infection compared to 2 patients in the hospitalized group. 18 patients in the ambulatory group had only month and year specified for the date of SARS-CoV-2 infection compared to 3 patients in the hospitalized group.*

*^f^In total, 80 patients had results available. One patient in the ambulatory group had lung transplantation because of severe bronchiectasis before the SARS-CoV-2 infection and was converted to absence of bronchiectasis.*

*^g^In total, 80 patients had results available. The analysis was performed with Mann-Whitney U test. One patient in the ambulatory group had bronchiectasis in the right middle lobe and was categorized as 1. One patient in the ambulatory group had basal cylindric bronchiectasis in left lobe and one high resolution computed tomography that mentioned bronchiectasis in lingula and was categorized as 1. One patient in the hospitalized group had computed tomography during severe Covid-19 infection and the bronchiectasis severity level of 1 was based on that diagnostic imaging. One patient in the hospitalized group was diagnosed with bronchiectasis but no grading was available from the medical records, and the mean group value of 1 was imputed for the patient.*

*^h^The 6 patients in the late ambulatory group with no diagnosed Covid-19 infection were excluded from the statistics. One patient in the late ambulatory group had sinusitis specified as superinfection and was reported as no superinfection.*

*^i^Analysis of the 11 patients who were reported with superinfection with Mann-Whitney U test.*

*^j^In total, 34 patients with bronchiectasis were analyzed, since 4 bronchiectasis patients had no known Covid-19 infection and were excluded.*

*^k^In total, 61 patients had results available. 40 patients were included in Welch’s t test.*

*^l^In total, 48 patients had results available. 33 patients were included in the Welch’s t test.*

*^m^In total, 30 patients had results available. 21 patients were included in the Welch’s t test.*

*^n^In total, 29 patients had results available. 20 patients were included in the Welch’s t test.*

**Supplementary Table 3** **Logistic regression analysis of factors associated with hospitalization due to SARS-CoV-2 infection in patients with XLA in the whole observation period**

|  | Univariate analysis  OR (95% CI) | *p* value | Multivariable analysis^a^  OR (95% CI) | *p* value | Multivariable analysis^b^  OR (95% CI) | *p* value | Multivariable analysis^c^  OR (95% CI) | *p* value | Multivariable analysis^d^  OR (95% CI) | *p* value |
| --- | --- | --- | --- | --- | --- | --- | --- | --- | --- | --- |
| Higher age | 1.43 (0.46 – 4.41) | *0.54* |  |  |  |  |  |  |  |  |
| Bronchiectasis | 6.77 (1.59 – 28.91) | ***<0.01*** | 8.39 (1.55 – 45.52) | ***0.01*** | 11.76 (2.21 – 62.87) | ***<0.01*** | ***–*** |  | 10.22 (1.92 – 54.29) | ***<0.01*** |
| Lower CD4^+^ T cell counts | 2.00 (0.49 – 8.18) | *0.33* |  |  |  |  |  |  |  |  |
| Lower NK cell counts | 1.28 (0.32 – 5.17) | *0.72* |  |  |  |  |  |  |  |  |
| Lower IgG levels | 9.62 (2.57 – 36.04) | ***<0.01*** | – |  | 11.16 (2.50 – 49.76) | ***<0.01*** | 13.57 (2.94 – 62.70) | ***<0.01*** | 7.68 (1.63 – 36.19) | ***<0.01*** |
| Early infection date | 11.16 (2.86 – 43.54) | ***<0.01*** | 8.98 (2.04 – 39.62) | ***<0.01*** | 7.55 (1.62 – 35.23) | ***0.01*** | 14.63 (3.11 – 68.76) | ***<0.01*** | ***–*** |  |
| No Covid-19 vaccination | 5.48 (1.64 – 18.31) | ***<0.01*** | 11.73 (2.33 – 59.09) | ***<0.01*** | ***–*** |  | 16.49 (3.01 – 88.58) | ***<0.01*** | 2.02 (0.43 – 9.61) | *0.38* |

*^a^Odds ratio adjusted for the two covariates high age and low IgG levels before infection.*

*^b^Odds ratio adjusted for the two covariates high age and no Covid-19 vaccination before infection.*

*^c^Odds ratio adjusted for the two covariates high age and bronchiectasis.*

*^d^Odds ratio adjusted for the two covariates high age and early date of infection.*

*The variables have been reanalyzed into dichotomous variables; under or median value for all study participants versus above median value for all study participants. Lower IgG levels were categorized as < 8 g/L and early date of infection was up until November 2021, to keep the same cut-off as in the main analysis.*
